# Early PSA Decline Predicts Survival Outcomes in Metastatic Castration-Resistant Prostate Cancer Treated with Androgen Receptor Pathway Inhibitors: A Retrospective Single-Center Study

**DOI:** 10.3390/medicina62061181

**Published:** 2026-06-18

**Authors:** Engin Hendem, Mehmet Zahid Koçak, Oguzhan Yıldız, Mustafa Korkmaz, Muhammed Muhiddin Er, Murat Araz, Mehmet Artac, Melek Karakurt Eryılmaz

**Affiliations:** 1 Department of Medical Oncology, Erzincan Mengücek Gazi Eğitim ve Araştırma Hastanesi, 24100 Erzincan, Turkey; 2 Department of Medical Oncology, Necmettin Erbakan University, 42090 Konya, Turkey; mehmetzahidkocak@hotmail.com (M.Z.K.); oguzhan.yildiz.07@gmail.com (O.Y.); zaratarum@yahoo.com (M.A.); mehmetartac@yahoo.com (M.A.); drangelkarakurt@hotmail.com (M.K.E.); 3 Department of Medical Oncology, Süleyman Demirel University, 32260 Isparta, Turkey; dr.musstafa@gmail.com; 4 Department of Medical Oncology, Konya City Hospital, 42020 Konya, Turkey; muhiddiner09@gmail.com

**Keywords:** metastatic castration-resistant prostate cancer, PSA decline, androgen receptor pathway inhibitors, abiraterone, enzalutamide, survival outcomes

## Abstract

*Background and Objectives*: Metastatic castration-resistant prostate cancer (mCRPC) remains a clinically heterogeneous condition despite ongoing advances in systemic treatment. Androgen receptor pathway inhibitors (ARPIs), including abiraterone acetate and enzalutamide, have been associated with improved clinical outcomes; however, early identification of patients deriving limited benefit continues to be challenging. Prostate-specific antigen (PSA) kinetics may serve as a practical indicator of treatment response over time. This study aimed to examine the prognostic significance of achieving a ≥50% reduction in PSA levels at three months in patients with mCRPC treated with ARPIs in routine clinical practice. *Materials and Methods*: In this retrospective single-center study, patients with mCRPC who received abiraterone acetate or enzalutamide between February 2015 and March 2024 were included. Patients were stratified according to PSA decline at three months (≥50% vs. <50%). Progression-free survival (PFS) and overall survival (OS) were estimated using the Kaplan–Meier method and compared with the log-rank test. Prognostic variables were subsequently examined using univariate and multivariate Cox proportional hazards models. *Results*: A total of 60 patients were included. At three months, 44 patients (73.3%) achieved a ≥50% decline in PSA levels. Patients reaching this level had longer PFS and OS than those with <50% decline, and the differences between groups were statistically significant. In multivariate analysis, early PSA decline remained significantly associated with improved survival outcomes. *Conclusions*: A ≥50% decline in PSA levels at three months represents a simple and clinically meaningful indicator of treatment response in patients with mCRPC receiving ARPIs. Early PSA kinetics may assist in timely risk stratification and closer clinical monitoring in routine clinical practice.

## 1. Introduction

Prostate cancer is one of the most commonly diagnosed malignancies among men worldwide and remains a major cause of cancer-related morbidity and mortality [1]. Despite advances in early detection and screening strategies, a substantial proportion of patients either present with advanced disease or later develop metastatic progression. Androgen deprivation therapy (ADT) continues to represent the cornerstone of initial systemic treatment in advanced prostate cancer; however, nearly all patients eventually progress to metastatic castration-resistant prostate cancer (mCRPC), a condition characterized by disease progression despite castrate levels of testosterone and generally associated with unfavorable survival outcomes [2,3].

Over the past decade, the therapeutic landscape of mCRPC has evolved considerably, largely driven by the introduction of novel systemic agents targeting androgen receptor signaling pathways. Androgen receptor pathway inhibitors (ARPIs), including abiraterone acetate and enzalutamide, have demonstrated clear survival benefits across both chemotherapy-naïve and post-chemotherapy settings and are now widely used in routine clinical practice [4,5,6]. These agents act through distinct mechanisms to suppress androgen receptor activity and delay disease progression. Even so, responses are not uniform across patients. While some individuals achieve sustained benefit, others experience limited response or early progression, sometimes within the first months of therapy. This variability remains clinically relevant and continues to raise the need for early and practical indicators of treatment efficacy [7]. In practice, however, such variability is frequently encountered and often complicates treatment decisions.

Prostate-specific antigen (PSA) remains central to the monitoring of prostate cancer and is still the most widely used biomarker in routine clinical practice. Beyond reflecting disease burden, its dynamic changes during treatment have attracted increasing attention. PSA kinetics, rather than isolated values, may provide a more nuanced picture of tumor biology and treatment sensitivity [8]. In clinical settings, changes in PSA levels after treatment initiation are often interpreted alongside radiological and clinical findings. Several studies have suggested that PSA declines during therapy are associated with improved progression-free survival (PFS) and overall survival (OS), indicating that PSA response may carry meaningful prognostic value [9,10,11,12]. Accordingly, a decline of ≥50% from baseline has been widely accepted as a clinically relevant response criterion and incorporated into evaluation frameworks proposed by the Prostate Cancer Working Group [11]. In routine care, PSA trends are often interpreted together with clinical judgment rather than in isolation.

At the same time, interpretation of PSA response is not entirely straightforward. Definitions of response vary across studies, and the timing of PSA assessment is not always consistent. In addition, patient populations differ considerably, which contributes to variability in reported outcomes. In many studies, PSA responses have been evaluated at different time points, ranging from a few weeks to several months after treatment initiation, making comparisons between studies more difficult and limiting their applicability to daily clinical practice [13,14].

Furthermore, a substantial portion of the available evidence is derived from clinical trial populations, which are often highly selected and may not fully reflect real-world clinical settings. In contrast, patients encountered in routine practice typically present with greater heterogeneity, including differences in disease burden, comorbid conditions, and prior treatment exposure. These factors may influence both treatment response and survival outcomes [7,15]. Within this context, identifying a simple and reproducible early biomarker that can be easily integrated into clinical practice becomes particularly important. A predefined time point—such as three months after initiation of ARPI therapy—may offer a practical window for early response assessment and risk stratification.

In reality, treatment decisions are rarely straightforward in this setting. Given the increasing complexity of treatment sequencing in mCRPC and the expanding range of available therapeutic options, timely identification of patients who are unlikely to benefit from ongoing ARPI therapy carries clear clinical importance. Early recognition of suboptimal response may allow clinicians to adapt treatment strategies more effectively, whether through closer monitoring or earlier transition to alternative therapies. Therefore, the present study aimed to evaluate the prognostic significance of achieving a ≥50% decline in PSA levels at three months in a retrospective single-center cohort of patients with mCRPC treated with ARPIs, reflecting routine clinical practice.

## 2. Materials and Methods

### 2.1. Study Design and Patient Selection

This retrospective single-center cohort study was conducted at the Department of Medical Oncology, Necmettin Erbakan University Meram Faculty of Medicine, Konya, Türkiye. A total of 60 consecutive patients with metastatic castration-resistant prostate cancer (mCRPC) who received abiraterone acetate or enzalutamide between February 2015 and March 2024 were included in the study.

The diagnosis of mCRPC was established based on disease progression despite castrate levels of testosterone, supported by biochemical, radiological, and/or clinical evidence.

Inclusion criteria were:

(1) histologically confirmed prostate adenocarcinoma;
(2) documented metastatic castration-resistant disease;
(3) treatment with abiraterone acetate or enzalutamide;
(4) available baseline PSA measurement;
(5) available PSA assessment at approximately 3 months after treatment initiation.

Exclusion criteria included:

(1) absence of 3-month PSA data;
(2) undocumented castrate testosterone levels at ARPI initiation;
(3) concurrent second primary malignancy.

**Figure 1:**
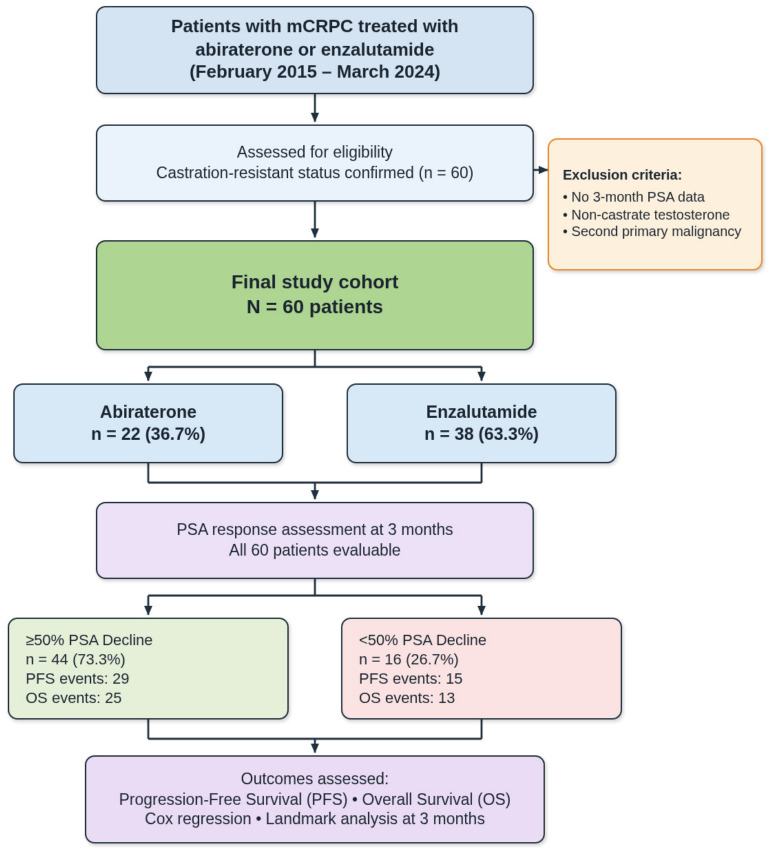
Flowchart illustrating patient selection, treatment allocation (abiraterone acetate or enzalutamide), assessment of 3-month PSA response, and survival analyses in patients with metastatic castration-resistant prostate cancer (mCRPC).

Clinical and demographic data, including age, ECOG performance status, metastatic burden, Gleason score, treatment history, and laboratory parameters, were retrospectively collected from institutional medical records. A flowchart of patient selection is presented in Figure 1.

### 2.2. Treatment and Response Assessment

Treatment decisions were made in routine clinical practice without a predefined study protocol. Patients received either abiraterone acetate (1000 mg daily with prednisone 5 mg twice daily) or enzalutamide (160 mg daily) according to standard clinical practice.

Follow-up was performed at regular intervals. PSA levels were measured at baseline and reassessed approximately three months after treatment initiation as part of routine monitoring.

PSA response was assessed according to the relative change in PSA levels at three months compared with baseline. Patients were classified into two groups: ≥50% PSA decline from baseline (good response, *n* = 44) and <50% PSA decline (poor response, *n* = 16).

The ≥50% threshold was selected based on established Prostate Cancer Clinical Trials Working Group (PCWG) recommendations and has been widely used in landmark clinical trials evaluating androgen receptor pathway inhibitors. Previous studies have demonstrated that a ≥50% PSA decline is associated with improved clinical outcomes, supporting its use as a clinically meaningful indicator of treatment response in patients with mCRPC.

### 2.3. Study Endpoints

The primary endpoints were progression-free survival (PFS) and overall survival (OS).

PFS was defined as the time from ARPI initiation to documented disease progression (radiographic or clinical) or death from any cause.

OS was defined as the time from ARPI initiation to death from any cause.

Patients who were alive without evidence of progression at the time of analysis were censored at the date of last follow-up.

### 2.4. Statistical Analysis

Baseline characteristics were summarized using descriptive statistics. Continuous variables were expressed as median with interquartile range (IQR), and categorical variables as frequencies with percentages.

Survival curves were estimated using the Kaplan–Meier method and compared using the log-rank test. Cox proportional hazards regression was used to estimate hazard ratios (HRs) with 95% confidence intervals (CIs). Univariable analysis was performed for all candidate prognostic factors. Variables with *p* < 0.10 in univariable analyses were considered for multivariable modeling.

Collinearity between candidate variables was assessed before multivariable analysis. Because tumor volume and risk group demonstrated complete overlap in the study cohort (Cramér’s V = 1.000), only tumor volume was included in the final multivariable model.

To address potential immortal time bias associated with PSA response assessment at 3 months, a landmark analysis was performed using a predefined 3-month landmark.

All statistical analyses were performed using IBM SPSS Statistics version 26.0 (IBM Corp., Armonk, NY, USA). A two-sided *p*-value < 0.05 was considered statistically significant.

### 2.5. Ethical Considerations

The study was carried out in accordance with the Declaration of Helsinki and approved by the local institutional ethics committee. Due to the retrospective design, the requirement for informed consent was waived.

## 3. Results

### 3.1. Patient Characteristics

A total of 60 patients with metastatic castration-resistant prostate cancer (mCRPC) were included in the study. The median age was 68 years (range: 49–83).

At the third-month evaluation, 44 patients (73.3%) achieved a ≥50% decline in prostate-specific antigen (PSA) levels, whereas 16 patients (26.7%) showed a decline of <50%.

Baseline demographic and clinical characteristics according to PSA response are summarized in Table 1. The majority of patients had high-risk disease, high tumor volume, and a Gleason score ≥ 8 at diagnosis.

No statistically significant differences were observed between patients achieving a ≥50% PSA decline and those with a <50% decline in terms of age (*p* = 0.482), ECOG performance status (*p* = 0.583), history of local surgery (*p* = 0.386) or radiotherapy (*p* = 0.398), presence of de novo metastasis (*p* = 0.583), Gleason score (*p* = 0.141), tumor volume (*p* = 0.378), or risk classification (*p* = 0.378).

Similarly, no significant differences were found regarding the type of ARPI treatment (abiraterone vs. enzalutamide, *p* = 0.347), the line of treatment, or metastatic distribution, including the presence of visceral metastases (*p* = 0.199) (Table 1).

The proportion of patients achieving a ≥50% PSA decline was 76.3% among those treated with enzalutamide and 68.2% among those treated with abiraterone, without a statistically significant difference between treatment groups.

### 3.2. PSA Kinetics

PSA kinetics according to 3-month PSA response groups are presented in Table 2. Baseline PSA values were comparable between groups (*p* = 0.967), whereas 3-month PSA values were significantly lower among patients achieving a ≥50% PSA decline (2.3 vs. 16.0 ng/mL, *p* = 0.004). Median PSA reduction was −83.7% versus −12.9% (*p* < 0.001). PSA nadir characteristics did not differ significantly between groups.

### 3.3. Survival Outcomes

Patients achieving a ≥50% PSA decline at three months demonstrated significantly improved survival outcomes compared to those with a <50% decline.

**Figure 2:**
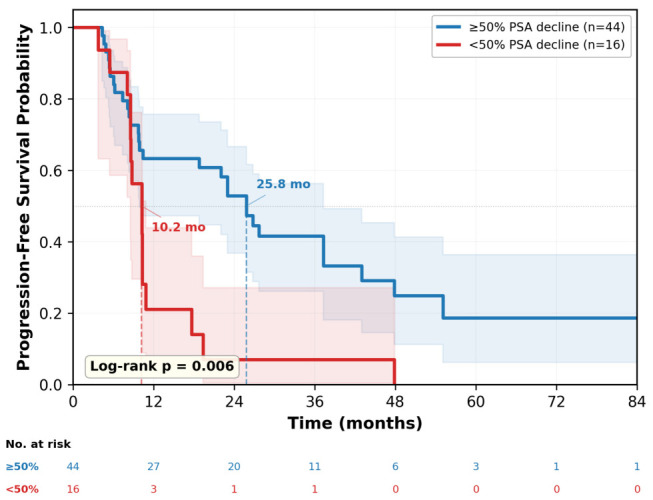
Kaplan–Meier Curve for Progression-Free Survival According to 3-Month PSA Response.

Median progression-free survival (PFS) was significantly longer in the ≥50% PSA decline group (25.8 months) compared with the <50% decline group (10.2 months) (log-rank *p* = 0.006; Figure 2).

**Figure 3:**
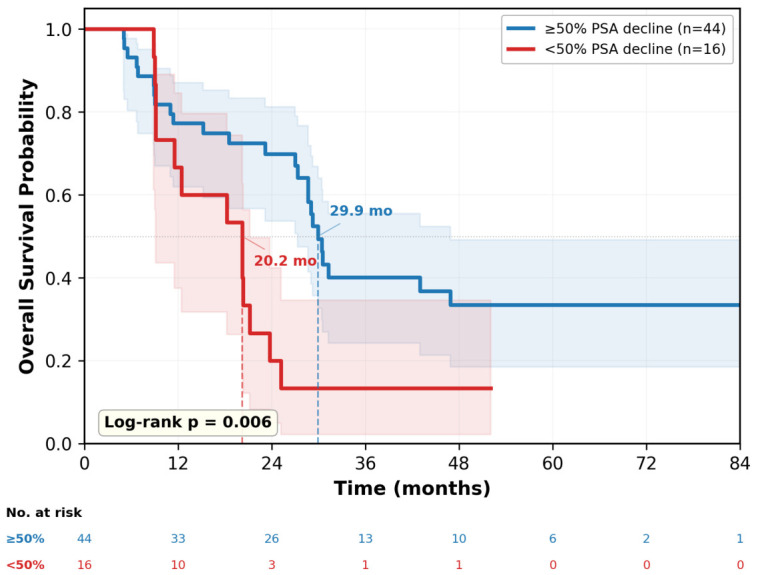
Kaplan–Meier Curve for Overall Survival According to 3-Month PSA Response.

Similarly, overall survival (OS) was significantly prolonged in patients with a ≥50% PSA decline (29.9 months) compared with those with a <50% decline (20.2 months) (log-rank *p* = 0.006; Figure 3).

### 3.4. Prognostic Factors

Univariate and multivariate Cox regression analyses for overall survival are presented in Table 3.

In univariate analysis, low tumor volume, low-risk disease, and PSA decline at three months were significantly associated with overall survival. In multivariable Cox regression analysis for overall survival, achieving a ≥50% PSA decline at three months remained a significant prognostic factor (HR = 0.444, 95% CI 0.220–0.896; *p* = 0.0235). Low tumor volume was also significantly associated with improved overall survival (HR = 0.242, 95% CI 0.083–0.701; *p* = 0.009). In contrast, Gleason score and the type of ARPI treatment (abiraterone vs. enzalutamide) were not significantly associated with survival outcomes.

### 3.5. Landmark Analysis

To address potential immortal time bias associated with defining PSA response at 3 months, a predefined landmark analysis was performed using 3 months after ARPI initiation as time zero. No PFS or OS events occurred before the landmark time point. Consistent with the primary analyses, patients achieving a ≥50% PSA decline demonstrated significantly longer landmark PFS and landmark OS compared with those achieving <50% decline (Table 4).

## 4. Discussion

This study examined the prognostic significance of early prostate-specific antigen (PSA) reduction in patients with metastatic castration-resistant prostate cancer (mCRPC) undergoing treatment with androgen receptor pathway inhibitors (ARPIs). The findings indicated that a reduction of ≥50% in PSA levels at three months correlated with markedly enhanced progression-free survival (PFS) and overall survival (OS). This connection remained significant after controlling for potential confounders, suggesting that early PSA kinetics may offer clinically relevant and prognostic insights. From a pragmatic standpoint, these preliminary alterations frequently represent the primary quantifiable indicators that clinicians evaluate when establishing an initial assessment of therapy efficacy.

Although ARPIs have clearly reshaped the therapeutic landscape of mCRPC, treatment responses are far from uniform. While some patients experience durable disease control, others progress relatively early despite appropriate therapy. This variability is not always predictable at baseline. Traditional prognostic indicators—such as disease burden, prior lines of therapy, or performance status—certainly contribute to risk assessment, yet they do not fully reflect the evolving biology of the disease once treatment has started. In daily practice, clinicians rarely rely on a single parameter; instead, early PSA changes are interpreted alongside imaging findings and clinical evolution, often serving as a practical anchor in this multidimensional evaluation.

The relationship between early PSA decline and favorable outcomes has been described in several prior studies. Facchini et al. reported that early PSA response to abiraterone was associated with prolonged survival [15], while Bosso et al. demonstrated similar findings in patients treated with enzalutamide [16]. Subsequent analyses have reinforced these observations, consistently showing that early PSA reductions correlate with improved outcomes across different ARPI-treated populations [17,18,19,20]. Importantly, these associations are not merely statistical; they likely reflect underlying tumor sensitivity to androgen receptor–targeted therapy. Still, it should be kept in mind that the magnitude and timing of PSA response can vary, and its interpretation may not always be straightforward in individual patients. The choice of a ≥50% PSA decline threshold was based on its widespread adoption in clinical trials and routine clinical practice. This threshold has been incorporated into the Prostate Cancer Clinical Trials Working Group (PCWG) recommendations and has been consistently associated with improved clinical outcomes across multiple studies of androgen receptor pathway inhibitors. Although alternative PSA response cutoffs have been explored, the ≥50% threshold remains the most extensively validated and clinically recognized marker of treatment response in mCRPC.

One aspect that deserves emphasis is the use of a fixed three-month time point for PSA assessment in the present study. Unlike studies that evaluate PSA responses at heterogeneous intervals, this approach mirrors routine clinical decision-making more closely. In many centers, the first meaningful reassessment of treatment response occurs within this timeframe. It is at this stage that clinicians often begin to question whether the chosen therapy is delivering the expected benefit. In that sense, the three-month evaluation is not arbitrary; it reflects a point where clinical intuition and objective data begin to converge.

More complex PSA-based metrics, such as PSA KELIM, have been proposed to improve prognostic accuracy and have shown encouraging results [18]. However, their applicability in everyday clinical settings may be limited by the need for repeated measurements and more elaborate calculations. By contrast, a simple threshold such as a ≥50% PSA decline is easy to apply and interpret. This simplicity should not be underestimated. In real-world oncology practice, decision-making often occurs under time constraints, and easily accessible markers tend to have greater clinical impact than more sophisticated but less practical tools.

Biologically, the absence of early PSA decline might suggest intrinsic or acquired resistance to ARPI therapy. Several mechanisms have been implicated including androgen receptor splice variants, receptor amplification, intratumoral androgen synthesis, and activation of alternative signaling pathways [4,21,22]. These mechanisms allow tumor cells to escape from androgen receptor blockade and continue to proliferate. However, the clinical relevance of these mechanisms is likely to vary between patients and the relative contribution of each mechanism to resistance is not fully understood. In practice, these molecular processes are seldom directly measurable, which highlights the relevance of surrogate markers like PSA kinetics.

Our results are of particular clinical relevance in the context of an increasingly complex treatment landscape. Treatment sequencing has become more nuanced by the availability of several therapeutic options, including chemotherapy, radioligand therapy and targeted agents. Early identification of those patients unlikely to benefit from continued ARPI therapy may help avoid unnecessary delays in transitioning to alternative therapies. But it is rare that treatment is decided upon based on PSA alone. Rather, PSA trends are integrated with clinical judgment, patient condition, and radiological findings. Even so, an early unfavorable PSA trajectory often raises concern and may prompt closer monitoring or earlier reassessment.

Our findings also support the general idea that dynamic biomarkers should be incorporated in clinical decision-making. Baseline characteristics are static and provide a snapshot, whereas early treatment-related changes provide a more immediate reflection of tumor behavior. That is a key distinction. Real time treatment response is provided by dynamic markers such as PSA decline and this may therefore lead to more individualized management strategies. These markers together with well-characterized clinical variables may improve risk stratification and allow more flexible treatment approaches [23,24,25,26,27].

However, there are multiple limitations to consider in interpreting these findings. First, the retrospective design of the study inherently limits the study in aspects such as possible selection bias and inability to establish causality. Second, the relatively small sample size may limit the subgroup analyses. Third, the single-center setting could limit generalizability to broader populations. In addition, molecular data were not available and we could not further investigate resistance mechanisms. Finally, differences in follow-up intervals and imaging practices reflect real-world conditions but may introduce heterogeneity in outcome assessment.

Despite these limitations, the study provides insights that are directly applicable to routine practice. The use of real-world data enhances external validity, and the focus on a simple, widely available biomarker increases clinical relevance. In many situations, clinicians must make decisions based on incomplete or evolving information. In this context, early PSA dynamics can serve as a useful and pragmatic guide.

Collectively, these results indicate that patients with mCRPC who are receiving ARPI therapy may benefit from clinically relevant insights into their treatment response resulting from early PSA decline. In routine practice, these early alterations can provide an additional perspective during the initial evaluation period, complementing radiological and clinical assessments. Simultaneously, the biological heterogeneity of the disease and the variability in individual responses necessitate that PSA dynamics be interpreted with caution and within the broader clinical context. In order to determine the most effective method of incorporating this parameter into personalized treatment strategies and to further elucidate the role of early PSA kinetics in guiding therapeutic decisions, additional prospective and multi-center studies are required.

The results were further supported by a predefined 3-month landmark analysis designed to reduce potential immortal time bias. Landmark PFS and OS analyses yielded findings consistent with the primary analyses, supporting the robustness of the observed association between early PSA decline and survival outcomes.

### Limitations

This study has several important limitations that should be considered when interpreting the results.

1. **Retrospective single-center design:** The study is inherently limited by its retrospective nature and single-center setting, which may limit the generalizability of the findings.
2. **Small sample size:** With 60 patients, the statistical power for detecting moderate effect sizes is limited. The multivariable model included only two variables to maintain an adequate events-per-variable ratio, which precluded adjustment for additional potential confounders.
3. **Heterogeneous imaging protocols:** Disease progression was assessed using conventional imaging (CT and bone scan) according to routine clinical practice. The absence of standardized imaging intervals and the non-use of next-generation imaging (e.g., PSMA-PET/CT) may have led to imprecision in determining progression dates.
4. **No molecular or genomic data:** We did not have access to molecular profiling (e.g., homologous recombination deficiency status, AR-V7 expression) that could provide additional prognostic information and identify subgroups with differential benefit.
5. **Potential residual confounding:** Despite multivariable adjustment, unmeasured confounders (e.g., prior treatment details, comorbidity burden, socioeconomic factors) may have influenced the observed associations.
6. **Single-line treatment only:** This study evaluated PSA responses during a single line of ARPI therapy. The prognostic significance of PSA responses in sequential ARPI settings was not assessed.

Therefore, external validation in larger multicenter cohorts is warranted to confirm the generalizability of these findings.

## 5. Conclusions

In this exploratory retrospective cohort study of 60 mCRPC patients, a ≥50% PSA decline at 3 months of ARPI treatment was associated with significantly longer progression-free and overall survival. This association remained evident after multivariable adjustment and was supported by a predefined 3-month landmark analysis. These findings suggest that early PSA response may serve as a practical prognostic marker in mCRPC, although the results should be considered hypothesis-generating given the study limitations. Validation in larger prospective multicenter cohorts is warranted before routine clinical implementation.

## Figures and Tables

**Table 1:** Baseline characteristics according to 3-month PSA decline (≥50%).

3-Month PSA Decline ≥ 50%	YES (*n*: 44, 73.3%)	NO (*n*: 16, 26.7%)	*p*-Value	SMD
**Age** ≥65 <65	16 (%36.4) 28 (%63.6)	5 (%31.3) 11 (%68.7)	0.482	0.108
**ECOG** 0–1 2–3	28 (%63.6) 16 (%36.4)	10 (%62.5) 6 (%37.5)	0.583	0.024
**Local Surgery** Yes No	9 (%20.5) 35 (%79.5)	2 (%12.5) 14 (%87.5)	0.386	**0.216**
**Local Radiotherapy** Yes No	8 (%18.2) 36 (%81.8)	4 (%25) 12 (%75)	0.398	0.166
**De novo Metastasis** Yes No	28 (%63.6) 16 (%36.4)	10 (%62.5) 6 (%37.5)	0.583	0.024
**Gleason Score at Diagnosis** 6 7 8 9 10	3 (%6.8) 11 (%25) 15 (%34.1) 9 (%20.5) 6(%13.6)	0 (%0) 3 (%18.8) 4 (%25) 2 (%12.5) 7 (%43.8)	0.141	
**Gleason Score at Diagnosis** <8 ≥8	14 (%31.8) 30 (%68.2)	3 (%18.8) 13 (%81.2)	0.256	0.034
**Tumor Volume** low high	12 (%27.3) 32 (%72.7)	3 (%18.8) 13 (%81.2)	0.378	**0.204**
**Risk Group** low high	12 (%27.3) 32 (%72.7)	3 (%18.8) 13 (%81.2)	0.378	**0** **.204**
**ARPI Treatment** Abiraterone Enzalutamide	15 (%34.1) 29 (%65.9)	7 (%43.8) 9 (%56.2)	0.347	0.199
**First-line Metastatic Treatment** ADT ADT + Docetaxel ADT + Abiraterone ADT + Enzalutamide	20 (%45.5) 19 (%43.2) 2 (%4.5) 3 (%6.8)	10 (62.5%) 5 (%31.3) 1 (%6.2) 0 (%0)	0.521	
**Line of Abiraterone/Enzalutamide** 1 2 3	4 (%9.1) 27 (%61.4) 13 (%29.5)	0 (%0) 11 (%68.8) 5 (%31.3)	0.457	
**Sites of Metastasis** Bone only Soft tissue Visceral Visceral + Bone	33 (%75) 6 (%13.6) 3 (%6.8) 2 (%4.5)	14 (%87.5) 2 (%12.5) 0 (%0) 0 (%0)	0.559	
**Visceral Metastasis** Yes No	5 (%11.4) 39 (%88.6)	0 (%0) 16 (%100)	0.199	

Abbreviations: SMD, standardized mean difference. SMD values > 0.20 were considered indicative of potential imbalance between groups. Risk group and tumor volume were perfectly collinear (Cramér’s φ = 1.000). *p*-values were retained from the original analyses (chi-square test, Fisher’s exact test, or Mann–Whitney U test, as appropriate).

**Table 2:** PSA kinetics according to 3-month PSA response groups.

Variable	Overall (*n* = 60)	≥50% Decline (*n* = 44)	<50% Decline (*n* = 16)	*p*
Baseline PSA, ng/mL	23.0 (8.3–59.8)	26.0 (7.0–59.0)	23.0 (10.0–68.8)	0.967
3-month PSA, ng/mL	6.0 (0.1–20.1)	2.3 (0.0–13.5)	16.0 (9.3–45.8)	0.004
PSA change, %	−75.9 (−93.8–−51.7)	−83.7 (−99.4–−72.0)	−12.9 (−43.5–−7.0)	<0.001
PSA nadir reached, *n* (%)	21 (35.0)	17 (38.6)	4 (25.0)	0.377
Time to PSA nadir, months	3.0 (1.0–3.0)	3.0 (1.0–3.0)	3.5 (1.0–6.8)	0.596

Values are presented as median (IQR) unless otherwise indicated. *p*-values were calculated using the Mann–Whitney U test for continuous variables and Fisher’s exact test for categorical variables. Time to PSA nadir was calculated only among patients who achieved a PSA nadir.

**Table 3:** Univariate and multivariate Cox regression analyses for overall survival.

Variable	Univariate HR (95% CI)	Univariate *p*-Value	Multivariate HR (95% CI)	Multivariate *p*-Value
Gleason (<8/≥8)	1.003 (0.496–2.030)	0.992	—	—
Low vs High Tumor Volume	0.233 (0.082–0.662)	0.006	0.242 (0.083–0.701)	0.009
Low vs High Risk	0.233 (0.082–0.662)	0.006	—	—
3-month PSA Decline (≥50% vs. <50%)	2.386 (1.194–4.766)	0.014	0.444 (0.220–0.896)	0.0235
Abiraterone vs Enzalutamide	1.520 (0.794–2.910)	0.206	1.038 (0.518–2.080)	0.915

**Table 4:** Landmark analysis at 3 months: survival outcomes by PSA response.

Outcome	Group	N	Events	Median (Months)	95% CI	HR	95% CI (HR)	Log-Rank *p*
Landmark PFS	≥50% PSA decline	44	29	22.8	6.9–34.3	Ref	—	0.004
	<50% PSA decline	16	15	7.2	5.5–7.8	2.57	1.33–4.96	
Landmark OS	≥50% PSA decline	44	25	26.9	24.3–43.9	Ref	—	0.012
	<50% PSA decline	16	13	17.2	6.1–18.2	2.37	1.19–4.73	

Note: No patients had PFS or OS events before the 3-month landmark; the landmark cohort comprises all 60 patients. Time zero was reset to 3 months from ARPI initiation. HR = hazard ratio (univariable Cox); <50% PSA decline vs. ≥50% PSA decline (reference). Log-rank χ^2^: PFS = 8.21, OS = 6.29.

## Data Availability

The original contributions presented in this study are included in the article. Further inquiries can be directed to the corresponding author.

## Abbreviations

The following abbreviations are used in this manuscript:

mCRPC	metastatic castration-resistant prostate cancer
PSA	prostate-specific antigen
ARPI	androgen receptor pathway inhibitor
ADT	androgen deprivation therapy
PFS	progression-free survival
OS	overall survival
ECOG	Eastern Cooperative Oncology Group
CI	confidence interval
HR	hazard ratio
